# An infrared 3D scanning device as a novel limb volume measurement tool in breast cancer patients

**DOI:** 10.1186/s12957-020-02043-y

**Published:** 2020-10-27

**Authors:** Bernadette N. White, Iris M. Lu, LeslieAnn S. Kao, J. Brandon Dixon, Michael J. Weiler, Nathan D. Frank, Jill Binkley, Preeti Subhedar, Joel Okoli, Karen Buhariwalla, Adriana Suarez-Ligon, Sheryl G. A. Gabram-Mendola

**Affiliations:** 1 Emory University School of Medicine and Grady Memorial Hospital Avon Comprehensive Breast Center, Atlanta, GA USA; 2 Georgia Institute of Technology, Atlanta, GA USA; 3 LymphaTech Inc., Atlanta, GA USA; 4 TurningPoint Breast Cancer Rehabilitation, Atlanta, GA USA; 5 Morehouse School of Medicine, Atlanta, GA USA; 6 Cherokee Breast Center Northside Hospital, Canton, USA; 7 Rutgers New Jersey Medical School, Newark, NJ USA

**Keywords:** Breast cancer rehabilitation, Lymphedema, Limb volume measurement, 3D technology

## Abstract

**Abstract:**

**Background:**

Lymphedema is a common complication of breast cancer treatment that affects one in five breast cancer survivors, yet there is no reliable method to detect lymphedema in the subclinical range. The objective of this study was to determine the feasibility and reliability of using an infrared 3D scanning device (ISD) as a peri-operative limb volume measurement tool.

**Methods:**

Fifteen patients were analyzed based on inclusion criteria. Peri-operative measurements were obtained using tape measure and an ISD. Volumes were calculated using a standard algorithm for tape measure and a custom algorithm for ISD measurements. Linear regression models were used to assess ISD and tape measurement volume and circumference correlation. One-way ANOVA was used to compare change in percent difference at set time points post-operatively (2–3 weeks, 4–6 weeks, and 7–12 weeks) for both ISD and tape measure. *t* tests for unequal variances with the Bonferroni correction were performed among these groups.

**Results:**

There is a positive linear correlation (*R*^2^ = 0.8518) between absolute volume measurements by the ISD and tape measure. Analyses over 2–10 weeks post-operatively showed that the ISD was able to detect volume changes in both the unaffected and the affected arm. Furthermore, the affected arm tended to have a greater increase in volume in the majority of patients, indicating these patients could be at risk for lymphedema.

**Conclusions:**

Technology utilizing infrared 3D scanners can reliably measure limb volume pre- and post-treatment similarly to tape measure in a small sample of patients. Further research using 3D scanning technology with a longer follow up is warranted.

## Introduction

Breast cancer is the most common cancer affecting women in the USA [1], and breast cancer-related lymphedema (BCRL) is the most common physical impairment affecting breast cancer survivors [2, 3]. Published data reports a wide range of 2–65% of breast cancer survivors suffering from lymphedema [3–6]. Lymphedema is a risk factor for other adverse events, including recurrent infection, functional impairments, decreased quality of life, and psychological distress [7–11]. Unfortunately, BCRL is frequently diagnosed after progressing to later stages and causing significant morbidity [12]. The pathology of lymphedema is partially to blame; patients begin to develop symptoms of arm heaviness, swelling, tightness, and achiness when arm volume increases 5–9.9%, and clinicians cannot reliably diagnose lymphedema until 10–20% swelling [13–16]. A reliable limb volume measurement tool that detects swelling in the asymptomatic range of 3–5% is essential to identify BCRL early when it can be managed most effectively and progression can be reduced or avoided [3, 16].

The Prospective Surveillance Model (PSM) was proposed in 2012 [17] to provide a standardized method to screen survivors for physical impairments due to breast cancer. Major goals of the PSM included surveillance for common physical impairments, education to reduce risk, early identification of impairments, and introduction of rehabilitation and exercise interventions to treat lymphedema. Pre-operative assessment had previously been shown to improve the monitoring and detection of lymphedema [16], but no standardized method had been proposed until the PSM. A follow-up study implementing the PSM found that 15% of the patients who underwent breast cancer surgery developed lymphedema, and there was a need for individualized rehabilitation intervention in one-third of all patients who underwent breast cancer surgery [18]. The PSM was resource intensive, requiring frequent evaluation by physical therapists and demonstrated the need for reliable, affordable, easily reproducible, and user-friendly screening tools [18].

Commercial infrared sensors, such as the Xbox Kinect IR sensor (Microsoft, Inc, Redmond, USA), offer a method to fulfill these requirements [19]. Infrared scanning is quick, inexpensive, hygienic, has no radiation exposure, and is easily accessible to both clinics and patients; it has been utilized previously to generate volume measurements in pediatric patients with vascular malformations [20] and in patients with filarial lymphedema [21]. In these prior studies, measurement data was analyzed using proprietary software (LymphaTech, Atlanta, GA, USA) to produce a digital three-dimensional reconstruction of the patient’s upper body from which a volume measurement of each limb was generated. This software system has been previously validated in the breast cancer population and found to generate reliable upper body measurements [22]. The Kinect IR sensor combined with this proprietary software has also previously been optimized by measuring more than 100 patients at risk for lymphedema at TurningPoint Breast Rehabilitation Center in Atlanta, Georgia [23]. Tape measurement was chosen in the current study as the comparison tool because it is the most-studied, most familiar and accessible to clinicians, and is reproducible if proper anatomical landmarks are established [23, 24].

The objectives of this study were (1) to determine how well the ISD volume calculations correlated with the tape measurements and (2) to determine the feasibility of implementing an ISD as a routine, easy-to-use clinical limb volume measurement tool. This study is the first known in the literature to apply the Kinect IR sensor as an ISD in a clinical setting for breast cancer patients, pre- and post-operatively at an urban, safety net hospital.

## Materials and methods

This study was performed in the Avon Foundation Comprehensive Breast Center at Grady Memorial Hospital, a safety net hospital in Atlanta, Georgia. Enrollment started in June 2016 and concluded in June 2017. Chart review was performed prior to each breast surgical oncology clinic to identify patients who fit the inclusion and exclusion criteria for the study.

*Inclusion criteria*: stage 0–III breast cancer and patients receiving upfront surgery or returning for surgery after neoadjuvant therapy and before radiation therapy. Exclusion criteria included prior history of breast cancer, prior axillary surgery or radiation, and inability to stand for at least 5 min. These criteria were established to capture patients receiving breast and axillary surgery and to exclude patients who already had any stage of lymphedema.

**Fig. 1:**
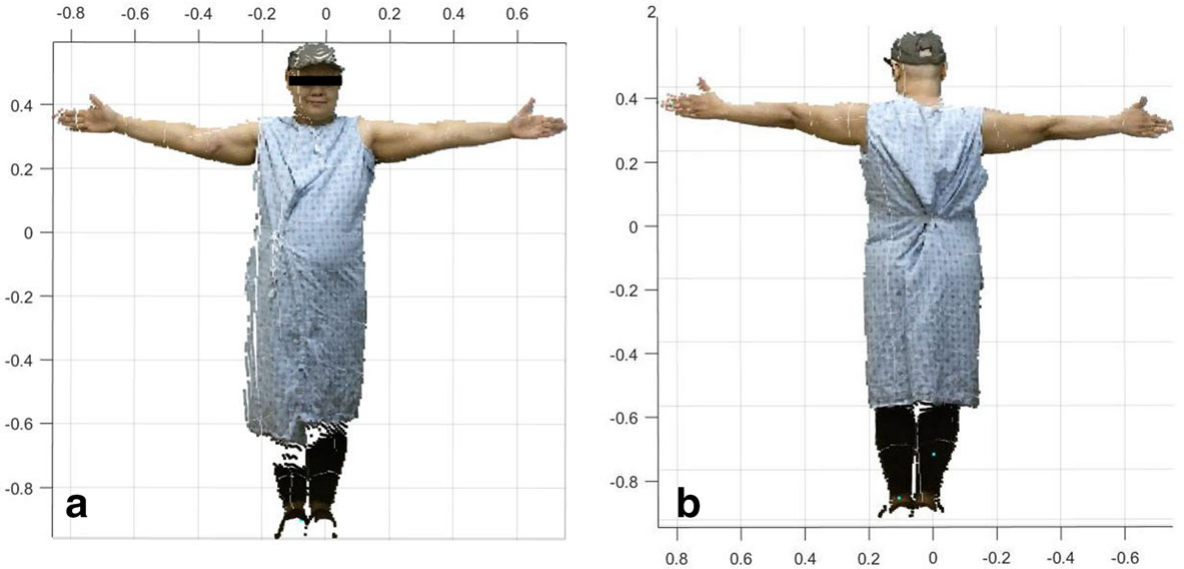
**a** Point cloud 3D reconstruction–front. **b** Point cloud 3D reconstruction–back

Following consent, the patient was led into a private room to complete the measurements. The examination room contained an examination table for performing tape measurements as well as the ISD, a Kinect IR sensor connected to a computer running proprietary software for limb volume measurement. Patients removed bracelets and watches and rolled up sleeves to the shoulder to ensure that the arm was not obstructed. Arms were first measured circumferentially using tape measurements every 4 cm from the ulnar styloid of the wrist to the axilla. Next, the patient stood approximately 6 ft. from the sensor with legs shoulder-width apart, head looking forward in neutral position, and the arms in proper positioning (Fig. 1) to obtain 4 measurements (front, back, right side, and left side), each side taking about 30 s. Patient arm positioning had to fit two criteria: (1) at least 75° from the trunk for anterior and posterior measurements, and 45 degrees for each side measurement, and (2) no more than 5° of shoulder flexion anteriorly. As a guideline to assist the measuring researcher, a 3D reconstruction of the patient’s body appeared on the connected computer, which turned from red to blue when the patient was within the appropriate parameters. The measuring researcher provided guidance to ensure that the patient was positioned within the defined parameters.

After obtaining the first pre-operative measurement, the patient was measured at subsequent post-operative visits at least 1 and up to 3 times. In addition to measurements, patient data was obtained and recorded in accordance with best clinical practices, including weight, height, body mass index (BMI), past medical and surgical history, and diagnosis and treatment plan. All data obtained was linked to a unique patient identifier code and stored for analysis with the measurements from the ISD and tape measurement data.

Tape measurement volumes were calculated using the truncated cone method, which takes the circumferential measurements and outputs the volume. Volumes from the ISD were determined using a custom algorithm designed to calculate a volume measurement from the scans generated by the device (Fig. 1a, b). For both the tape measurements and the 3D measurements, the volume was calculated from wrist to axilla every 4 cm, sparing the hand. Analysis by the GT team included a determination of the quality of the scans, from low to high quality, as well as the limb volume calculation. If scan quality was low, then a volume calculation could not be performed by the algorithm.

In order to differentiate between normal post-operative swelling, which can persist for several weeks following surgery, and BCRL, which typically presents months to years after treatment [25–28], percent differences in volumes between affected and unaffected arms were calculated with each 3D measurement. Percent difference was defined by convention as the difference between affected and unaffected limb volumes divided by the unaffected limb volume. A change greater than 5% after the first 6 weeks post-operatively was considered concerning for possible subclinical lymphedema.

Linear regression was utilized to assess the ISD and tape measurement correlation and the operator variation. A Bland-Altman Plot was used to determine the agreement between the volumes gathered by the ISD and tape measure circumferences; the difference in measurement between the two modalities was plotted against the average. One-way ANOVA was used to compare change in percent difference at set time points post-operatively (2–3 weeks, 4–6 weeks, and 7–12 weeks) for both ISD and tape measure. *t* tests for unequal variance were used to assess ISD measurement percent difference between these set post-operative time points. An alpha value was set to 0.05, therefore, any *p* value of < 0.05 was considered significant.

## Results

There were 106 eligible patients screened via chart review during the study period. Twenty-three patients were recruited and consented; 2 withdrew prior to completing the study. Seventeen patients had pre-operative scans from which volumes could be calculated, while 6 had scans that could not be calculated due to clothing covering portions of the arm. Fifteen patients had at least one post-operative scan, with the other 6 patients being lost to follow-up. Table 1 shows the demographics of the patients who completed the study. All patients had either SLNB or axillary dissection, with 6 out of the 15 participants undergoing axillary dissection.

**Table 1:** Participant characteristics (*n* = 15)

Demographic characteristics	Count (%)
Age	
< 40	2 (13)
41–65	9 (60)
> 65	4 (27)
Race	
White	1 (6)
African American	14 (93)
Comorbidities	
Hypertension	9 (60)
Diabetes mellitus	4 (27)
Tobacco use	2 (13)
Body mass index (kg/m^2^)	
< 18.5 (underweight)	0
18.5–24.9 (normal)	4 (27)
25–29.9 (overweight)	5 (33)
> 30.0 (obese)	6 (40)
**Breast cancer history**	
Stage	
0	1 (6)
1	4 (27)
2	7 (47)
3	3 (20)
Breast cancer type	
DCIS	1 (7)
Invasive ductal	13 (87)
Invasive lobular	1 (7)
**Breast cancer treatment**	
Neoadjuvant chemotherapy	9 (60)
Surgery	
Partial mastectomy with SLNB^*a*^	3 (20)
Simple unilateral mastectomy with SLNB	5 (33)
Modified radical mastectomy	4 (27)
Bilateral mastectomy	3 (20)
Lymph nodes removed during surgery	
1–5	8 (53)
6–10	1 (7)
Axillary dissection	6 (40)

^a^*SNLB* sentinel lymph node biopsy

**Fig. 2:**
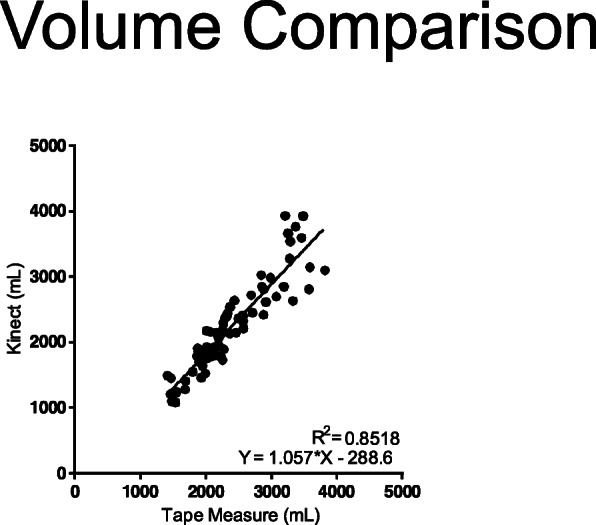
Linear regression curve for volume as measured by tape measurement and ISD

**Fig. 3:**
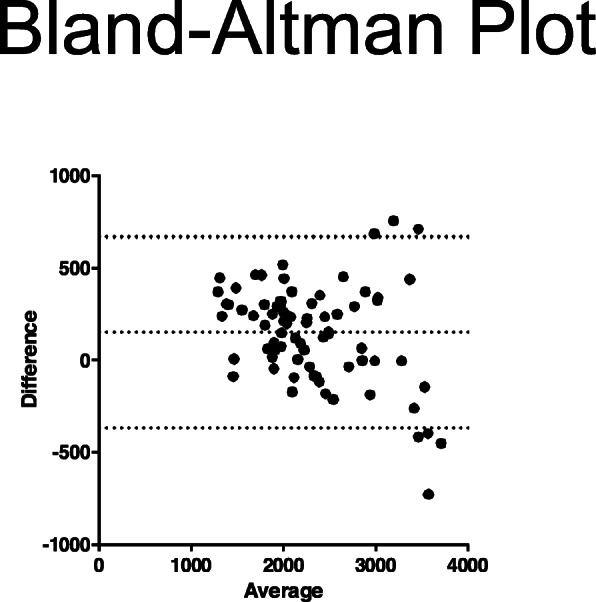
Bland-Altman plot of agreement between volumes gathered by ISD versus tape measure

Linear regression analysis of the 3D volume measurement and tape measurement volumes demonstrated a strong correlation (*R*^2^ = 0.8518), with the ISD tending to capture greater volumes than the tape measure technique (Fig. 2). A Bland-Altman plot indicating the degree of agreement between the two measurement techniques (Fig. 3) shows that larger arm volumes had less agreement between tape measure and the ISD.

**Fig. 4:**
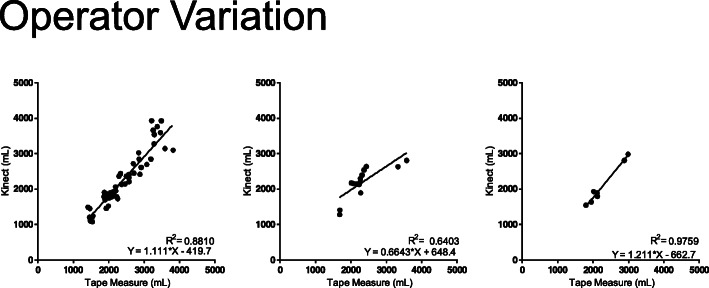
Inter-operator variability. Each graph represents an individual operator who took tape measurements and ISD scans

Looking at the individual operator variation (Fig. 4), the correlation of the volume gathered by the ISD and the tape measure circumference measurements was strong for two operators and moderate for one (*R*^2^ = 0.8810, 0.6403, 0.9759). Variability from graph to graph was more pronounced for the tape measurements.

Percent differences in volume changes at weeks 2–3, 4–6, and 7–12 were compared with one-way ANOVA for the ISD and tape measurement and were not found to be statistically significant. Additionally, *t* tests for unequal variance for the 3D scanner using these same time points were not found to be statistically significant.

## Discussion

From a clinical standpoint, this pilot study was successful in demonstrating a strong positive correlation with an *R*^2^ of 0.8518 between limb volume calculations obtained with a well-known and studied modality, circumferential tape measurements, and a novel infrared 3D scanning technology. The study also showed that symptomatic volume changes could be detected by the device, as one patient who was already undergoing treatment for lymphedema was found by the 3D scanner to have an increase in limb volume of about 15%.

The secondary goal of this study was to test how feasible the ISD would be as a routine screening instrument in the breast cancer clinic. We found that this device was easy to implement in the clinical setting and has distinct advantages compared to the other modalities currently in use. The infrared sensor that forms the backbone of this ISD uses similar technology to the Perometer, which is a highly sensitive optoelectrical scanner, and has been found to have a standard error of measurement (SEM) of 2.1% in one systematic review [29]. In comparison, in that same study, the SEM for tape measure was found to be 2.8%, and the SEM of water volumetry was 0.7%. Another method of evaluating for lymphedema is bioimpedance spectroscopy (BIS), which uses a low-frequency electrical current to detect the amount of fluid in the area of interest.

While studies have shown a lower incidence in lymphedema severity amongst breast cancer patients after treatment when undergoing early surveillance with BIS compared to a traditional referral model of care [30], there are no reports directly comparing the efficacy of early surveillance models leveraging BIS versus using limb volume changes. BIS has only been found to have modest correlation to Perometer, with one study finding *R* = 0.60 [31]. In addition, there have been reports that BIS has a false-negative rate of over 30% when compared to directly imaging lymphatic vessels using indocyanine green (ICG) lymphography [32]. There are also several emerging modalities that allow for the direct imaging of lymphatic vessels to detect abnormalities [33, 34]. These approaches will likely face challenges associated with widespread clinical adoption due to the need for expensive instrumentation or the injection of contrast agents.

An ISD offers similar accuracy to both tape measure and perometry, with our study finding a strong positive correlation to tape measure, and previous studies with larger cohorts of patients comparing volumes measured by this ISD and a Perometer, a precise tool to measure limb volumes, have found good correlation between the two (*R*^2^ = 0.8799) [35]. Importantly, the ISD costs a fraction of the price of Perometer (approximately $20,000) or of BIS (approximately $10,000), with the sensor averaging $400, while not sacrificing accuracy in detection of volume changes.

This study identified that the ISD accurately measures patients with body types ranging from normal weight to obese. The reasons for scans failing were improper body positioning and clothing that covered up a portion of the arms, not the circumference or volume of the patient’s arms. However, an important limitation of the device noted in this study is that there was more variability between the infrared measurements of larger limb volumes. In the future, problems with positioning may be resolved by utilizing hand supports to optimize arm position and minimize motion since the hands are not included as part of the arm volume calculation. This study also underscores the need for proper training of the staff measuring patients using these 3D scanners. Inter-operator variability was strong for two researchers and moderate for a third; adequate understanding of the parameters of the device will improve scan acquisitions and measurements and decrease variability between operators.

Other limitations of this study include the following: (1) it was conducted at a single institution, safety-net hospital, with a primarily Black population and so the results may not be representative of the general population; (2) it is a small sample size, making it difficult to draw conclusions about this ISD’s overall ability to measure volumes in a large cohort of patients [35]; and (3) the short duration of the study. Although volume changes were detected in this study, lymphedema develops in most breast cancer survivors over the course of years, and so longer follow-up with additional measurements is needed to determine if this ISD detects persistent and increasing volume changes. Finally, there is a lack of post-operative data for every patient enrolled in the study due to patient factors, time constraints of the study, and the limitations in the device discussed above. Thus, it was not possible to perform rigorous statistical analyses to test the ability of the ISD to detect volume changes over time as compared to tape measurement. However, since this was a pilot study intended to test feasibility, at this early stage this is an acceptable limitation.

Despite the study limitations, this feasibility study for the use of novel 3D scanning technology is an important step in lymphedema research. The patient data collected in conjunction with this study will help better identify patients at risk for lymphedema. Additionally, knowledge gathered in this study will be important for studying the pathophysiology and underlying causes of lymphedema and tissue swelling of all causes. The current study is an important step in assessing all types of patients in a quick and non-invasive manner.

## Conclusion and clinical practice points

This study was the first to demonstrate that a novel infrared 3D scanning technology, the Kinect IR sensor combined with volume calculation software, accurately measures limb volumes in breast cancer patients with comparable results to tape measure circumferential measurements. Further research using 3D scanning technology with a longer follow up is warranted.

- Infrared 3D scanning devices can measure limb volume, are easy to use, and are time efficient.
- Infrared 3D scanning device measurements and tape measure circumference measurements of limb volume in breast cancer surgical patients are strongly correlated.
- This pilot study supports the need for further research on the use of these infrared 3D scanning devices to assess for lymphedema in peri-operative breast cancer patients.

## Data Availability

The datasets used and/or analyzed during the current study are available from the corresponding author on reasonable request.
